# Analyzing the Impact of Binaural Beats on Anxiety Levels by a New Method Based on Denoised Harmonic Subtraction and Transient Temporal Feature Extraction

**DOI:** 10.3390/bioengineering11121251

**Published:** 2024-12-10

**Authors:** Devika Rankhambe, Bharati Sanjay Ainapure, Bhargav Appasani, Avireni Srinivasulu, Nicu Bizon

**Affiliations:** 1 Department of Computer Engineering, Vishwakarma University, Pune 411046, India; rankhambedevika@gmail.com; 2 School of Electronics Engineering, KKalinga Institute of Industrial Technology, Bhubaneswar 751024, India; bhargav.appasanifet@kiit.ac.in; 3 School of Engineering and Technology, Mohan Babu University (Erstwhile Sree Vidyanikethan Engineering College), Tirupati 517102, India; avireni@gmail.com; 4 Faculty of Electronics, Communication and Computers, Pitești University Center, National University of Science and Technology POLITEHNICA Bucharest, 110040 Pitesti, Romania

**Keywords:** binaural beats, anxiety, least mean square, electroencephalography, deep Q-network, Wiener filter

## Abstract

Anxiety is a widespread mental health issue, and binaural beats have been explored as a potential non-invasive treatment. EEG data reveal changes in neural oscillation and connectivity linked to anxiety reduction; however, harmonics introduced during signal acquisition and processing often distort these findings. Existing methods struggle to effectively reduce harmonics and capture the fine-grained temporal dynamics of EEG signals, leading to inaccurate feature extraction. Hence, a novel Denoised Harmonic Subtraction and Transient Temporal Feature Extraction is proposed to improve the analysis of the impact of binaural beats on anxiety levels. Initially, a novel Wiener Fused Convo Filter is introduced to capture spatial features and eliminate linear noise in EEG signals. Next, an Intrinsic Harmonic Subtraction Network is employed, utilizing the Attentive Weighted Least Mean Square (AW-LMS) algorithm to capture nonlinear summation and resonant coupling effects, effectively eliminating the misinterpretation of brain rhythms. To address the challenge of fine-grained temporal dynamics, an Embedded Transfo XL Recurrent Network is introduced to detect and extract relevant parameters associated with transient events in EEG data. Finally, EEG data undergo harmonic reduction and temporal feature extraction before classification with a cross-correlated Markov Deep Q-Network (DQN). This facilitates anxiety level classification into normal, mild, moderate, and severe categories. The model demonstrated a high accuracy of 95.6%, precision of 90%, sensitivity of 93.2%, and specificity of 96% in classifying anxiety levels, outperforming previous models. This integrated approach enhances EEG signal processing, enabling reliable anxiety classification and offering valuable insights for therapeutic interventions.

## 1. Introduction

Anxiety is one of the most common mental diseases and has a wide range of effects on a person’s life. There are several subtypes of it, such as panic disorder, social anxiety disorder (agoraphobia), obsessive–compulsive disorder (OCD), and generalized anxiety disorder (GAD) [1,2,3]. Physical symptoms include palpitations, tremors, trembling, and dyspnea. Other symptoms include difficulty focusing, dread of losing control, fear of public places, and specific fears related to situations, animals, or natural phenomena. Anxiety disorders represent a substantial burden on society, impacting approximately 7.3% of the global population. A recent study found that 1 in every 14 people may experience an anxiety disorder at some point in their lifetime. It has been connected to mental disorders such as depression and suicidal thoughts. It can also induce or worsen the signs and symptoms of other illnesses. This has led to the development of numerous anxiety treatments, many of which depend on musical stimulation.

One promising auditory stimulation technique is binaural beats, which are created when two slightly different frequencies are presented to each ear, producing the perception of a single tone with amplitude modulation [4,5]. Binaural beats have been shown to influence neural oscillations, enhancing phase synchronization in regions associated with emotional regulation and relaxation. For example, exposing each ear to frequencies of 400 Hz and 410 Hz results in the perception of a 405 Hz tone with a 10 Hz fluctuation. This phenomenon, known as binaural integration, reflects the brain’s ability to combine auditory information into a cohesive percept. Such transient event patterns involve discrete, repetitive alterations in neural activity, often triggered by external stimuli like binaural beats. These changes, occurring across various timescales, reflect the brain’s dynamic response to sensory inputs and emotional triggers [6,7]. Advancements in neural activity monitoring have shed light on the mechanisms underlying such phenomena. For instance, optoelectronic methods have enhanced the ability to observe and modulate neural circuits, enabling a more precise understanding of brain function. The heart–brain connection also offers valuable insights, revealing how systemic physiological signals such as heartbeats influence neural oscillations and, in turn, emotional states. Additionally, novel neural interfaces, including injectable fluorescent devices, have improved the resolution of neural data collection, allowing for cell-specific imaging and stimulation. These advancements underscore the importance of preserving spatial and temporal fidelity in neural signal analysis to understand the nuanced effects of interventions like binaural beats [8,9,10,11].

Prior research has demonstrated that binaural auditory beats begin in the superior olivary and brainstem nuclei, and then proceed to the reticular formation, from where they can be detected as a frequency following response (FFR) in the cerebral cortex [12,13]. The FFR is the propensity of the electrocortical activity of the brain to alter its relative power and synchronize its neural activity to the same frequency as an externally given stimulus. Numerous lines of evidence suggest that neuronal excitability is phase-locked and entrainment (phase-resetting) to the binaural beats. These processes modify the response gain, amplify neuronal responses, and ultimately align the rhythmic fluctuations in neuronal excitability so that the high excitability phases more closely coincide with the stimulus events [14].

Acquiring and processing the electroencephalographic (EEG) signal from each participant before and following the application of the binaural beat is necessary and legitimate to ascertain whether physical change was observed and how the resulting change affected the resultant signal [15,16,17]. Time-frequency, non-linear features, spectral (Nonparametrics, Parametrics, Coherence, etc.), temporal (Hjorth Parameters, Detrended Fluctuation Analysis), and other feature extraction techniques are commonly used by statisticians to track these changes. The variations in brain activity that occur across time are referred to as temporal dynamics. The way that brain activity changes in response to internal processes, sensory inputs, cognitive tasks, and external stimuli is captured by these changes, which can happen on a variety of timescales, from milliseconds to seconds, minutes, or even longer. Distinct temporal dynamics are displayed by various brain networks and areas, which represent the integration and synchronization of information processing. Additionally, preprocessing procedures like Independent Component Analysis (ICA) for artifact identification are frequently needed. Previous research investigated six distinct auditory signals (none, pure tone, classical music, 5 Hz, 10 Hz, and 15 Hz binaural beats), discovered statistically significant variations between them, and employed the delayed match to sample visual tasks to assess the effects of the signals on working memory. The metrics used for the investigation included time-frequency synchronization with the Phase-Locking Value (PLV), graphical network measures, and Connectivity Ratio (CR) [18,19].

There are several drawbacks to the current works, including the lack of a classifier-based analysis or processing phase. Furthermore, the effect of binaural beats on anxiety levels in EEG signals has not been extensively studied. Therefore, a unique model is crucial for the processing and analysis of EEG information, and it also allows for an effective examination of the effect of binaural beats on the levels of anxiety in EEG signals. The following are this paper’s main contributions:

- To eliminate the misinterpretation of the harmonic signal, a novel Intrinsic Harmonic Subtraction Network is introduced, which retains the relevant complicated neural dynamic component;
- To capture the temporal continuity of data in EEG, a novel Embedded Transfo XL Recurrent Network is introduced, which identifies and extracts the rapid oscillatory bursts or short-lived synchronization patterns that are hidden in the temporal continuity of the continuous EEG signal.

Considering the objectives mentioned above, this paper is structured in five sections as follows. After the introduction, Section 2 reviews the results recently published in the specialized literature. Section 3 details the new proposed method based on the Intrinsic Harmonic Subtraction Network and Embedded Transfo XL Recurrent Network. The comparative analysis of the results and the performances of the proposed method are highlighted in Section 4, and the conclusions are mentioned in Section 5.

## 2. Literature Survey

Garcia et al.’s [20] meta-analysis examined the impact of binaural beats on memory, attention, anxiety, and analgesia. The meta-regression results show that binaural beats were played without being covered up, suggesting that it is not necessary to mask them with white noise or pink noise in order to get comparable effects. Additionally, the results suggest that exposure to binaural beats prior to, during, and following a task produces superior results than exposure to task-related stimuli. That binaural-beat exposure was an effective method that also reduced anxiety and pain perception. The findings have indicated that binaural auditory beats affect memory, anxiety, focus, and pain perception in a passive, automatic way. The drawback of this work was the larger sample sizes for validating the meta-analysis. So far, the results of the meta-regression have been acquired in a theoretical form, which requires further comprehension for novel practical applications.

The goal of Munro et al.’s [21] proof-of-concept study was to examine the short-term effects of a binaural beat on tinnitus in order to assess whether more trials are required. The subjects were given two auditory stimuli: ocean waves with and without alpha frequency (8 Hz) and binaural beats. Arousal and tinnitus perception were measured before and following each sound stimulation using the Perceived Arousal Scale and tinnitus rating measures. The tinnitus rating scores marginally improved with sound. Some people showed more improvement with binaural beats than with just ocean waves. There are not enough data to support clinical research about the effects of the binaural beat with ocean sounds.

Rishika et al. [22] examined how adult brain waves are affected by binaural beat responses and how this helps to promote sleep. A low frequency of binaural beats applied near the subjects’ ears, and when the subjects fell asleep without the application of binaural beats, were the two conditions under which the head movement from the experiment was represented by angle coordinates based on the readings from the accelerometer and gyroscope sensors. Using real-time hardware experiment values, an application was created to distinguish between scenarios with and without binaural beats. The application experiment’s real-time hardware findings were used in a statistical analysis that ran the relevant tests to display the sleep pattern. This task requires more analysis of theta and delta brain waves and the effect of binaural beats on sleep patterns.

da Silva Junior et al. [23] delivered a 5 Hz binaural beat to six distinct volunteers in order to identify a significant shift in the subjects’ brainwaves both before and after the stimulation. This study used ten distinct stimulation sessions lasting twenty minutes each. The differences between the sessions were analyzed using a Multi-Layer Perceptron classifier, non-parametric testing, and Low-Resolution Brain Electromagnetic Tomography (eLORETA). Significant changes were shown by high Alpha eLORETA. Significant changes were observed with high Beta in both the eLORETA and MLP techniques. The reaction of MLP to theta brainwaves was substantial. The greater sample size required, control group, classifier application for validating binaural beat data, and efficiency of this approach were its drawbacks.

El-Ashmawi et al. [24] offered an automated model for bilateral negotiations that offered a solution based on chaos theory and the Chaotic Owl Search Algorithm (COSA), a modern version of the Owl Search Algorithm (OSA), a meta-heuristic algorithm. This algorithm was used to modify negotiation strategies for calculating offers during the negotiations. Two parties engaged in several rounds of negotiation to accomplish this. In this work, the COSA in optimization problems specifically, the negotiation process was performed for the first time. This approach needs further enhancement in multi-lateral negotiation. The multi-lateral negotiation can be modeled as a multi-bilateral negotiation with numerous negotiating parties and multiple negotiation processes occurring simultaneously. The integration of various chaotic maps is another direction that may have a significant impact on the search space for agreements.

Shamsi et al. [25] examined whether the Higuchi fractal dimension (HFD), which decreased the electroencephalogram (EEG) complexity by BB stimulation, is a dependable substitute for its equivalent linear measure (i.e., the relative power of the band in which the beat frequency lies) in the context of identifying brain entrainment. Furthermore, even for those electrodes of the temporal and parietal lobes that did not experience significant theta-band relative power variations, HFD led to generally higher classification accuracies and areas under the empirical ROC curve. Because this idea focused solely on the theta waves, it does not support the effect that binaural beats have on anxiety levels.

Alonso-Valerdi et al. [26] offered comparisons of sound therapy based on music, retraining, neuromodulation, and binaural sounds following psychological and neuro-audiology assessments. For sixty days, 76 participants with tinnitus received sound-based therapy. The approximation of the electrical neural activity’s entropy served as the foundation for the neuro-audiology evaluation. After that, a psychological assessment revealed that the best sound-based therapy for lessening tinnitus perception was usually retraining. Neuromodulation and binaural noises had remarkably comparable results in lowering anxiety, tension, and tinnitus perception. However, it is not recommended for individuals with anxiety problems.

Lee et al. [27] aimed to assess the effect of binaural beats on brain wave entrainment and suggest a safe, efficient adjunctive treatment for reducing insomnia symptoms. We recruited subjects from the population who had subclinical symptoms of insomnia, aged 20 to 59. For two times, two weeks prior to and two weeks following the BB intervention, quantitative electroencephalography was recorded. The relative theta power rose when BB-containing music was played. Following a fortnight of music intervention, there was an increase in theta power when listening to music using BB. Participants’ beta power decreased more after two weeks of music-listening with BB than after using devices with just audio when they listened to music in the lab. However, the results do not apply to everyone experiencing insomnia symptoms, and do not fully account for individual differences in reaction to binaural auditory beats.

Engelbregt et al. [17] investigated the effects of 40 Hz monaural beats (MB) and binaural beats (BB) on electroencephalography (EEG) and attention. Twenty-five first-year psychology students (11 males and 14 females) completed a Flanker task as their EEGs were being recorded for five minutes, while pink noise (PN), MB, and BB were presented. Regarding attention, as determined by the Flanker task, there were fewer erroneous replies in the BB condition than in the PN condition, but more false responses in the MB condition than in the PN condition. However, longer auditory beats and a more comprehensive battery of cognitive tests are necessary.

Leistiko et al. [28] used the attention network test (ANT), a hitherto unutilized task that evaluates three kinds of attention: alerting, orienting, and executive control, to examine the impact of gamma (40-Hz) BBs on attention. The ANT was conducted remotely by fifty-eight healthy people while they were exposed to 340-Hz beta bandgaps and a 380-Hz reference tone. Everyone filled out an anxiety rating form both before and after the exposure. Wilcoxon signed-rank tests were used to compare the performance of the BB and control groups on the ANT task (reaction time and mistake rates). However, it only investigated the effect of BB for a single exposure.

The above discussion has stated that [20] had limitations with larger sample sizes for verifying the meta-analysis, and the meta-regression results that were obtained up to that point were theoretical. Due to the lack of prior study, [21] does not have enough evidence supporting its claim that the binaural beat with ocean sounds can have a beneficial effect on clinical trials. Further research on Theta and Delta brainwaves as well as the impact of binaural beats on sleep patterns is needed [22]. A larger sample size, a control group, a classifier application for evaluating binaural beat data, and efficiency are required [23]. For [24], additional improvement in multilateral negotiation and integration of different chaotic maps is required, as these factors have a major influence on the area that needs to be searched for agreements. For [25], the research focused solely on the theta waves, and this theory does not support the effect that binaural beats have on anxiety levels. [26] is not recommended for individuals with anxiety problems and [27] does not fully account for individual differences in reaction to binaural auditory beats. In [27], longer auditory beats and a more comprehensive battery of cognitive tests are necessary, and [28] only investigated the effect of BB for a single exposure. Hence, a novel model has to be developed to analyze the effect of binaural beats on anxiety levels.

## 3. Denoised Harmonic Subtraction and Transient Temporal Feature Extraction

EEG data help in studying binaural beats’ effect on anxiety reduction by analyzing neural oscillations, connectivity patterns, and spectral power changes. Harmonics, often viewed as noise, can hinder analysis, particularly when they are unwanted artifacts in signal acquisition and processing. Hence, a novel Denoised Harmonic Subtraction and Transient Temporal Feature Extraction is proposed to overcome the limitations of the existing EEG signal processing approaches and improve the analysis of the impact of binaural beats on anxiety levels. Initially, a novel denoising technique, the Wiener Fused Convo Filter, is introduced in the preprocessing phase. The Convolutional AutoEncoder (CAE) captures spatial features in EEG signal components, like electrode-related noise, and estimates noise characteristics by comparing observed signals with pre-trained clean EEG data. The Wiener filter, integrated into the final processing layer of CAE, eliminates linear noise by minimizing the mean square error between the desired and observed noisy signals.

Current harmonics reduction methods often confuse actual brain rhythms with harmonic elements, potentially leading to the misinterpretation of neural activity. These methods rely on filters to identify and mitigate harmonics, yet they overlook resonant coupling effects and nonlinear neural activity summation, which is the result of interactions between various frequency oscillations within the neural populations in the brain (anxious multiple thinking). Hence, a novel Intrinsic Harmonic Subtraction Network is used, where the input layer employs the Hilbert–Huang Transform (HHT), which breaks down the denoised EEG data into instantaneous frequencies and intrinsic mode functions, providing non-linear oscillatory components; then, the CNN convolutional layers receive this deconstructed data and use them to record the resonant coupling effect and nonlinear summation in brain rhythm; lastly, the final output layer of the CNN introduces the Attentive Weighted Least Mean Square (AW-LMS) Algorithm, which iteratively updates the error signal coefficients to delete the other improper harmonic frequencies and assigns zero weight to the retained signal data.

Existing methods often fail to capture quasi-stable EEG topographies and fine-grained temporal dynamics, leading to inaccurate feature extraction. This is because transient events, such as rapid oscillatory bursts, are often hidden within the continuous EEG signal, eluding traditional techniques like clustering or topographic analysis. Hence, a novel Embedded Transfo XL Recurrent Network is introduced for capturing the temporal continuity of data in EEG, where the EEG data are first embedded with relative vector positional encoding to capture the temporal order of the brain rhythm pattern sequence. Next, the Transformer-XL Architecture receives the embedded EEG data and uses a dynamic allocative attention mechanism to identify the parameters in the signal patterns related to the brain’s transient events, such as frequency components, amplitude changes, shifts in the frequencies’ spectral power, and waveform morphology features like skewness and waveform area. This effectively captures the quasi-stable EEG topographies and the transient event patterns throughout the EEG data length. After detecting the features related to transient events, these data are sent to the Long Short-Term Memory (LSTM) unit which extracts these features using a dynamic context window that can expand or contract based on the presence of transient events.

Finally, these harmonic-reduced and temporal-feature-extracted EEG data are processed for classification using a novel cross-correlated Markov DQN, which determines the impact of different frequency bands (alpha, beta, theta, gamma, and delta) on the anxiety level. In this network, time delays between the signals of various binaural beats with respect to the frequencies of the binaural beats are found using a band cross-correlation analysis. Following this, the DQN incorporates Markov decision-making, enhancing the system’s ability to make decisions, and finally classifies the anxiety levels (normal, mild, moderate, and severe) using correlation analysis and the extracted features.

**Figure 1:**
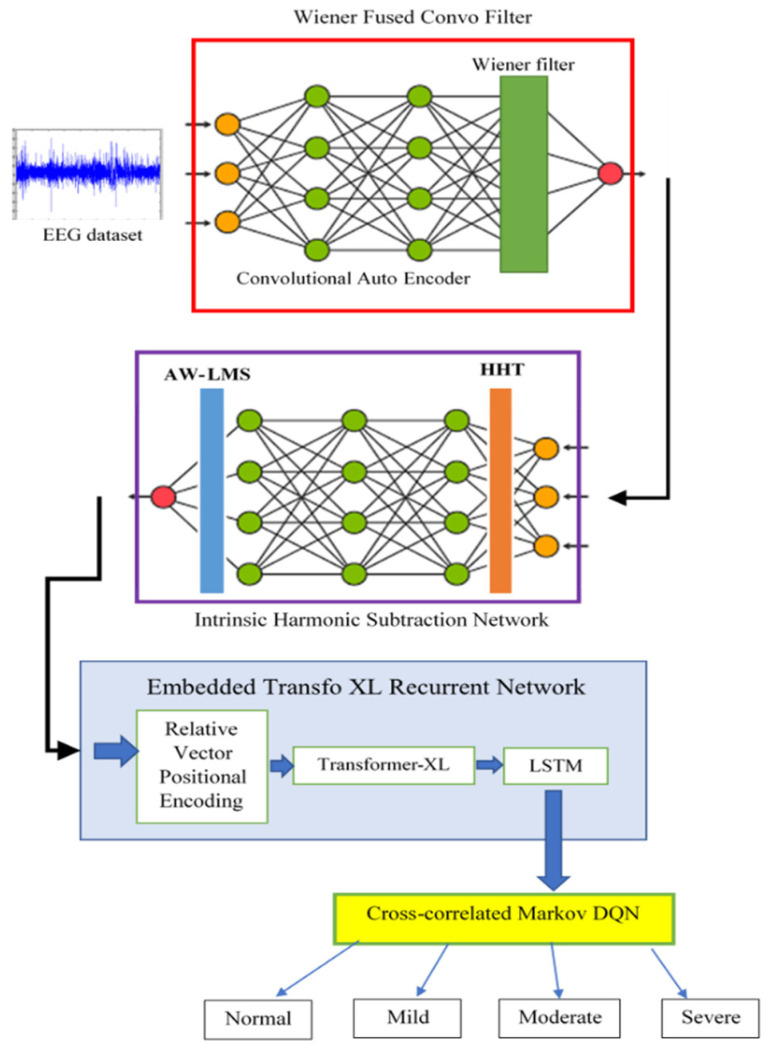
Block Diagram of the proposed system.

Figure 1 illustrates the block diagram of the proposed model. Initially, the data from the EEG dataset are sent to the Wiener Fused Convo Filter network, where CAE captures complex spatial features and the Wiener filter removes the linear noise components from EEG signals. Then, the denoised signal is sent to the Intrinsic Harmonic Subtraction Network, which uses the HHT and AW-LMS algorithm to eliminate the misinterpretation of the harmonic signal. Then, the temporal continuity of the data in the EEG is captured with the Embedded Transfo XL Recurrent Network, using relative vector positional encoding, Transformer-XL, and LSTM. Finally, the harmonic-reduced and temporal-feature-extracted EEG data are processed for classification using the cross-correlated Markov DQN, which classifies the anxiety levels (normal, mild, moderate, and severe) based on the extracted features and correlation analysis.

### 3.1. Dataset Description

The dataset used in this research consists of individuals, namely men and women, for four different states, such as normal, mild, moderate, and severe. In the dataset, the total data present are 945, of which 501 are male and 444 are female. The age group of the people selected is from 18–71. The EEG data were collected from the year 2011–2019. Here, 80% of the data are used for training and 20% of the data are used for testing. These selected data are sent to the Wiener Fused Convo Filter for pre-processing, which is explained in Section 3.2.

### 3.2. Wiener Fused Convo Filter

The pre-processing stage involves removing noise or artifacts that might interfere with the interpretation or processing of the EEG data. The proposed method introduces a novel denoising technique called the Wiener Fused Convo Filter with CAE, which is used for removing the noise from a signal. The Wiener filter is specifically designed to target linear noise components, while the CAE captures complex spatial features inherent in the EEG signals. EEG signals are inherently noisy due to various sources, including biological and environmental factors.

**Figure 2:**
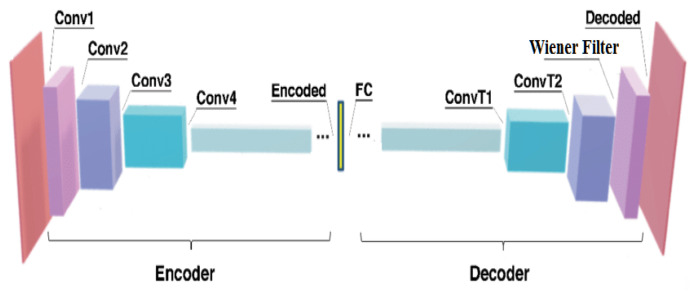
Wiener Fused Convo Filter.

Figure 2 shows the architecture of the Wiener Fused Convo Filter Network, which combines the Wiener filter and a CNN for pre-processing. Here, the Wiener filter is placed at the last layer of the CAE to eliminate the linear noise components in the EEG data. Complex spatial properties are captured by the CAE, which is trained on a dataset containing clean EEG data. During training, it learns to encode and decode these clean EEG signals, capturing their essential features. Once the CAE is trained, it is used to estimate the noise characteristics present in detected EEG signals. This process is explained in Equation (1), as follows:

(1) N=Y−X

where X represents the reconstructed EEG signals, N are the noise characteristics, and Y is the EEG signal. The detected EEG signals likely contain both the desired brain activity as well as various forms of noise. By comparing the detected signals with the pre-trained clean EEG data, the CAE estimates the noise characteristics present in the observed signals. The final processing layer of the CAE is then merged with a Wiener filter. The two stationary processes, y and $\hat{d}$, have a statistical relationship with one another. Since d and v are stationary random processes, the construction of the filter W minimizes the estimated mean square error. The Wiener filter is a classic signal processing technique used for noise reduction. The mean and variance surrounding each pixel are assessed by the Wiener filter. Equation (2) shows how to create the Wiener filter equation W by combining the Mean and Variance equations, as follows:

(2) $$W=\mu \left(B\right)+\frac{\sigma^{2}\left(B\right)-n^{2}}{\sigma^{2}\left(B\right)}\left(a-\mu \left(B\right)\right)$$

where μ is the mean, σ is the variance around each pixel, and $n^{2}$ is the noise variance. It operates by minimizing the mean square error between the desired signal (the clean EEG) and the observed noisy signal. The minimum mean square solution for the Wiener filter is given in Equation (3), as follows:

(3) $$W\left(n\right)=\frac{{\left|S\left(n\right)\right|}^{2}}{{\left|S\left(n\right)\right|}^{2}+{\left|V\;\left(n\right)\right|}^{2}}$$

where $W\left(n\right)$ is the Wiener filter, $S\left(n\right)$ is the signal from noisy observation, and $V\;\left(n\right)$ is the noise component. By leveraging the noise characteristics estimated by the CAE, the Wiener filter effectively removes the linear noise components from the observed EEG signals. To prevent the risk of attenuating meaningful EEG signals, the Wiener filter is explicitly tuned to avoid suppressing non-linear features critical for capturing neural activity. The subsequent stages handle non-linear noise and harmonics, complementing this approach. After this, the denoised signal is processed to eliminate the harmonics using a novel Intrinsic Harmonic Subtraction Network, which is explained in Section 3.3.

### 3.3. Intrinsic Harmonic Subtraction Network

The Intrinsic Harmonic Subtraction Network is designed to further process the denoised EEG signal to eliminate unwanted harmonics. Harmonics are multiples of the fundamental frequency of a signal and can introduce artifacts or distortions in EEG data. At the input layer, the denoised EEG data undergo decomposition using the HHT method, a powerful technique for analyzing non-linear and non-stationary data. This decomposition generates IMFs and instantaneous frequencies, providing detailed insights into the EEG signal’s non-linear oscillatory components. The decomposition process is mathematically expressed in Equation (4):

(4) $$H\left\{y\left(t\right)\right\}=\frac{1}{\pi }P\int_{-\infty }^{\infty }\frac{g\left(\tau \right)}{t-\tau }d\tau$$

where $y\left(t\right)$ are the denoised EEG data. This decomposition provides insights into the non-linear oscillatory components present in the EEG signal.

**Figure 3:**
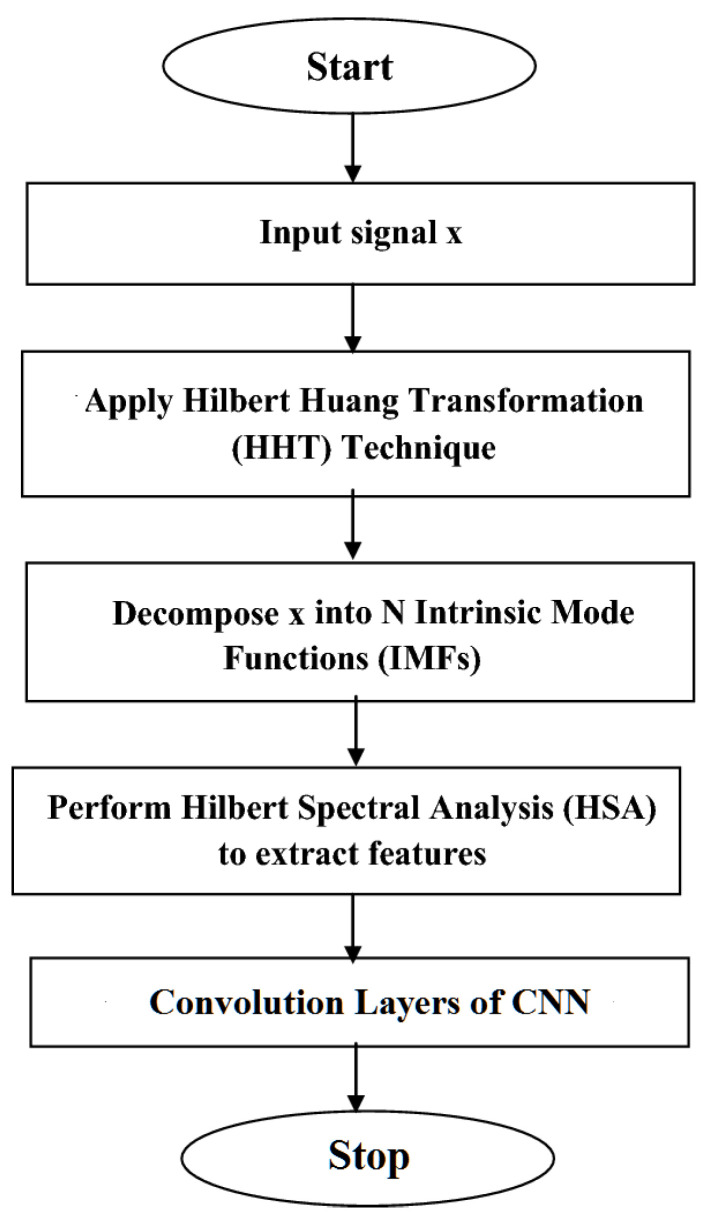
Flowchart of Hilbert–Huang transformation process of the proposed system.

Figure 3 demonstrates the flowchart of the HHT process of the proposed system. The signal represented by x is then fed into the system to be processed in the following phase. After that, the signal is subjected to the HHT approach, a non-stationary and nonlinear data analysis method. Subsequently, the signal is broken down into its intrinsic mode functions (IMFs), which are its core elements. Hilbert Spectral Analysis (HSA) is then used to extract features from the signal, providing a detailed representation of its frequency components across time. These features, derived from the HHT method, are rich in both time–frequency information, capturing the non-linear and non-stationary nature of EEG signals. To ensure compatibility with the CNN input expectations, the extracted features are first normalized and aligned. This preprocessing step standardizes the data, allowing the CNN to effectively process and learn from the temporal and spectral patterns inherent in the EEG signal. This alignment involves resizing and padding sequences to ensure uniformity, thereby preventing mismatches in sequence lengths between different EEG segments. Moreover, this process helps maintain the temporal relationships between the extracted features while adapting them to CNN’s receptive fields.

The HHT decomposition outputs non-stationary and non-linear components, which are essential for capturing the brain’s rhythmic behavior. The alignment process ensures that each time segment of the EEG data corresponds correctly across the channels and instances, preserving the essential temporal and spectral information necessary for the accurate analysis of brain dynamics related to anxiety levels.

After this alignment, the decomposed data from the HHT are sent to the convolutional layers of a CNN to capture spatial hierarchies in the data, making them suitable for processing complex EEG signals. Here, the CNN captures the nonlinear summation and resonant coupling effects present in the brain rhythm. These effects contribute to the complexity of EEG signals, and retaining them within the signal helps avoid the misinterpretation of neural activity.

At the final output layer of the CNN, an AW-LMS algorithm is introduced, which iteratively updates the error signal coefficients to subtract inappropriate harmonic frequencies. It assigns zero weight to the retained signal data, ensuring that only relevant neural activity is preserved. By subtracting inappropriate harmonic frequencies, the AW-LMS algorithm further enhances the denoising process and ensures that the resulting EEG signal is free from the misinterpretation of harmonic signals. The simple equation for AW-LMS is given in Equation (5), as follows:

(5) $$R=\sum_{i=1}^{N}{\left(y_{i}-w\left(u_{i}\right)\right)}^{2}\;$$

The CNN captures nonlinear summation and resonant coupling effects within the EEG signal. Finally, the AW-LMS algorithm eliminates inappropriate harmonic frequencies, ensuring that the resulting EEG signal accurately represents relevant neural dynamic components while eliminating the misinterpretation of harmonics.

**Figure 4:**
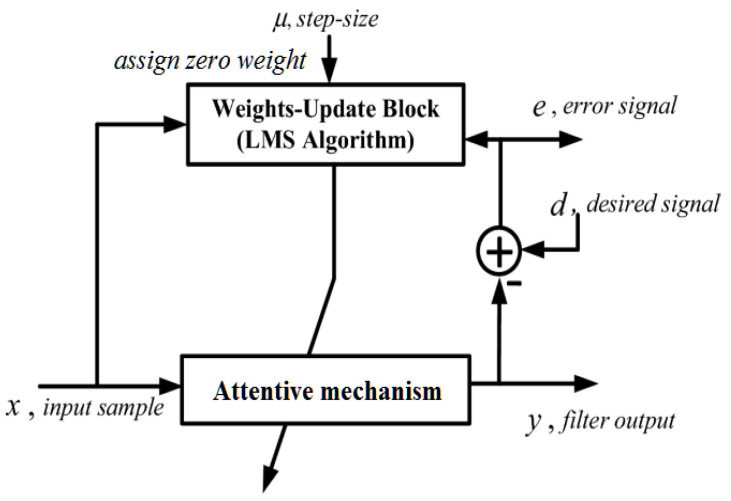
Attentive Weighted Least Mean Square (AW-LMS) algorithm of the proposed model.

Figure 4 displays the AW-LMS Algorithm of the proposed model, which employs the final output layer of the convolution layer of CNN. The system consists of several components to illustrate signal flow and weight updating. The input sample is fed into an attentive mechanism, which processes the signal, producing an output called y. The output is combined with a desired signal, d, to create an error signal, e, which minimizes the error. The error signal is then fed back into the Weights-Update Block (LMS Algorithm), which updates the filter’s weights based on the error signal. The step size parameter, μ, controls the rate of weight updates, affecting convergence speed and stability. The updated weights from the Weights-Update Block are then fed back to the attentive mechanism, completing the loop and allowing it to adapt its response based on the incoming signals and the desired output. With this novel denoising and Intrinsic Harmonic Subtraction Network, the misinterpretation of the harmonic signal is eliminated and the relevant complicated neural dynamic components are retained. The differentiation of noise-reduction roles ensures that redundancy is avoided while safeguarding the accuracy of neural activity representation. Then, a novel Embedded Transfo XL Recurrent Network is introduced for capturing the temporal continuity of data in EEG, which is explained in Section 3.4.

### 3.4. Embedded Transfo XL Recurrent Network

The Embedded Transfo XL Recurrent Network is designed to capture the temporal continuity of EEG data, focusing on detecting transient events and quasi-stable EEG topographies throughout the data. The combination of Transformer-XL and LSTM was specifically chosen for their complementary strengths in modelling temporal dependencies. Transformer-XL is adept at handling long-range temporal dependencies through its dynamic allocative attention mechanism and extended memory capabilities. This enables it to capture quasi-stable EEG topographies and long-term dependencies, such as gradual shifts in amplitude, spectral power changes, and waveform morphology over extended sequences. LSTM, on the other hand, excels at modelling local temporal dynamics and is particularly effective at capturing fine-grained, transient events like rapid oscillatory bursts or short-lived synchronization patterns. Using its dynamic context window, the LSTM adapts its receptive field to the significance and duration of these transient features.

The EEG data are initially embedded with relative vector positional encoding. This encoding technique helps capture the sequence of brain rhythm patterns in their temporal order. By encoding relative positional information, the network gains an understanding of the temporal relationships between different segments of EEG data. This process is explained below in Equation (6), as follows:

(6) $$z_{m}=\sum_{n=1}^{i}\alpha_{mn}\left(x_{n}W^{V}+a_{mn}^{V}\right)$$

Then, the embedded EEG data are fed into the variant of the Transformer architecture known as Transformer-XL, which employs a dynamic allocative attention mechanism, which allows it to attend to different parts of the input sequence effectively. In EEG analysis, the Transformer-XL Architecture is utilized to detect parameters related to transient events of the brain. These parameters include frequency components, amplitude changes, shifts in spectral power of frequencies, as well as waveform morphology features such as skewness and waveform area.

**Figure 5:**
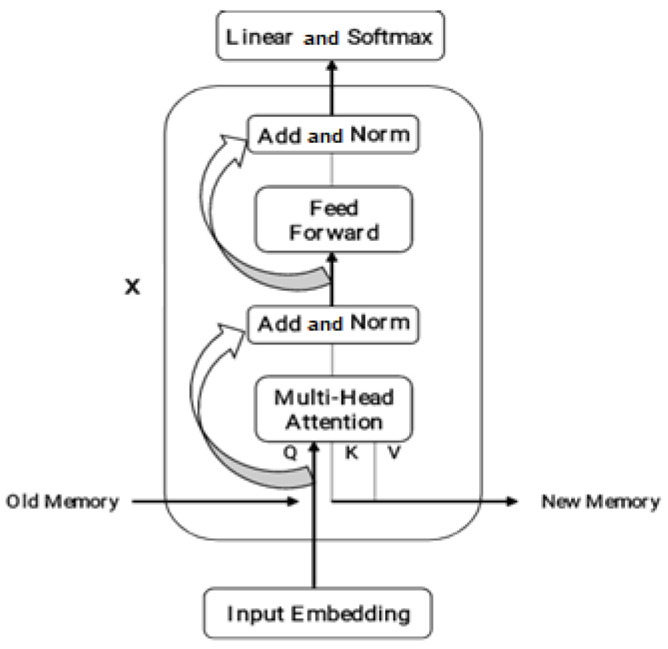
Schematic representation of a Transformer-XL.

Figure 5 shows the schematic representation of a Transformer-XL. The bottom input embedding transforms a fixed-size vector with “Add & Norm” for the residual link and layer normalization. Multi-head attention incorporates relative location encoding for self-attention, while feed-forward denotes the feed-forward neural network layer. The top output layer employs the Softmax function and linear transformation. x indicates the repetition of operations from “Add & Norm” to “Feed Forward”, with Q, K, and V representing attention mechanism elements, updating memory with old and new information. Formally, Transformer-XL redefines the equation that generates the values Q, K, and V of the attention mechanism as follows by Equations (7) and (8):

(7) $$Q=W_{Q}y$$

(8) $$K,V=W_{K,V}\left[SG\left(y_{t-1}\right)*y\right]$$

where [∗] refers to concatenation, SG refers to the stop-gradient function, and $y_{t-1}$ refers to the input to the attention mechanism from the previous sequence. By dynamically allocating attention, the network effectively captures quasi-stable EEG topographies and transient event patterns throughout the length of the EEG data. After detecting features related to transient events, the data are sent to the LSTM unit.

LSTM is a type of recurrent neural network (RNN) designed to handle sequence data. It is particularly effective in capturing long-term dependencies and patterns in sequential data. The LSTM unit extracts features from the data using a dynamic context window. This context window expands or contracts based on the presence of transient events in the EEG data. The mathematical depiction of the LSTM output of the $j^{th}$ cell at a time t is given in Equation (9), as follows:

(9) $$y^{ci}\left(t\right)=y^{out\;i}\left(t\right)h\left(s_{ci}\left(t\right)\right)$$

where $s_{ci}\left(t\right)$ is an internal state, $s_{c}$ is the scale, and h is the differentiable function.

**Figure 6:**
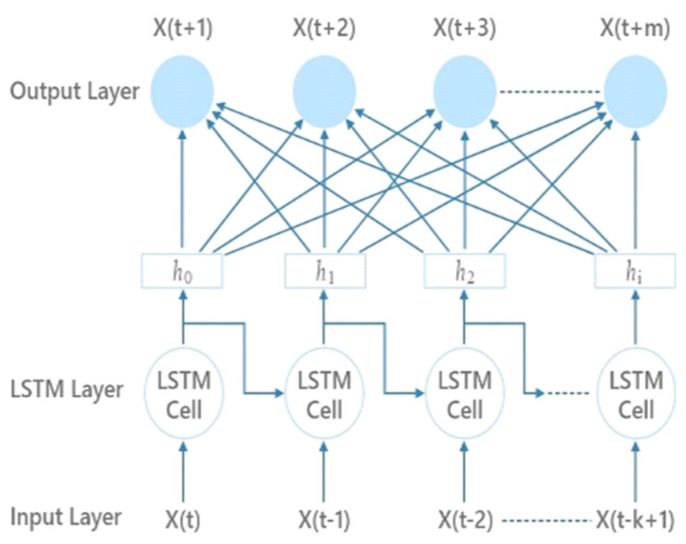
Long Short-Term Memory.

Figure 6 displays the LSTM of the proposed model. An input layer, hidden LSTM layers, and an output layer are the usual components of an LSTM structure. The model captures the intricate links between the auditory stimulus and the related changes in anxiety levels, because the LSTM layers are made to learn the long-term dependencies in the binaural beat data. By adapting its receptive field dynamically, the LSTM unit effectively captures rapid oscillatory bursts or short-lived synchronization patterns hidden within the temporal continuity of the continuous EEG signal.

The Transformer-XL provides the necessary context for transient event detection, while the LSTM refines this context by learning the dependencies and temporal patterns over longer sequences. The design prevents redundancy and overfitting by employing hierarchical feature extraction, where the outputs of the Transformer-XL and LSTM are merged at different levels of abstraction, rather than independently. The Transformer-XL captures the immediate, short-term patterns in the EEG data, while the LSTM captures the longer-term dependencies. This ensures that both the immediate and long-term temporal relationships in EEG signals are effectively captured without overlap. The merging of their outputs allows the model to efficiently integrate both immediate short-term patterns and long-term dependencies, ensuring a more robust feature extraction process. Hence, with this novel Embedded Transfo XL Recurrent Network, the rapid oscillatory bursts or short-lived synchronization patterns that are hidden in the temporal continuity of the continuous EEG signal are identified and extracted. Finally, these harmonic-reduced and temporal-feature-extracted EEG data are processed for classification using a novel cross-correlated Markov DQN, which is explained in Section 3.5.

### 3.5. Cross-Correlated Markov DQN

The cross-correlated Markov DQN network is designed for the classification of anxiety levels based on EEG data that have undergone harmonic reduction and temporal feature extraction. Unlike static classifiers like SVM, DQN captures complex temporal dependencies and nonlinear dynamics of EEG signals. It uses reinforcement learning and Markov decision-making to adapt to changing EEG states and model sequential data. DQN’s ability to analyze the interplay across different frequency bands enhances its accuracy, especially for anxiety-related neural events. Its iterative learning mechanism refines classification strategies over time. This dynamic approach effectively captures subtle patterns in anxiety levels by leveraging temporal dependencies in the EEG signals. To enhance this process, the processed EEG data undergo a band cross-correlation analysis, which identifies time delays between the signals of different frequency bands, such as alpha, beta, theta, gamma, and delta. Binaural beats are auditory illusions perceived when two slightly different frequencies are presented to each ear. Analyzing their cross-correlation helps understand the relationship between different frequency bands in the EEG signal.

The Markov decision-making process is a mathematical framework used to model decision-making in situations where outcomes are partially random and partially under the control of a decision-maker. The Markov Property asserts that only the current state, which contains all the relevant information from the past, is used to predict the future. Equation (10) is used to evaluate the Markov Property as follows:

(10) $$P\left[St+1\left|St]=P[St+1\;\right|S1,S2,S3\ldots \ldots St\right]$$

This equation indicates that the probability of the next state ($P\left[St+1\right]$), given the current state (St), is equal to the probability of the next state ($P\left[St+1\right]$), after taking into account all of the previous states (S1,S2,S3…).

The Markov decision-making process is incorporated into the DQN, which is a type of reinforcement learning algorithm used for decision-making tasks. It learns to map states to actions to maximize expected rewards. By integrating the Markov decision-making process into DQN, the decision-making capability of the system is improved. The DQN learns to make decisions based on both the current state of the EEG data and the expected future outcomes, taking into account the temporal dependencies and uncertainties in the data.

Anxiety levels can be determined by measuring brain activity, which is probably present in the input layer. Utilizing convolutional layers, pertinent features are extracted from the input data. After the features have been recovered, Fully Connected Layers uses them to learn how to forecast changes in anxiety levels in response to binaural beat stimuli. Predicting the user’s anxiety levels based on the physiological signals it receives and the effects of the binaural beats would be the DQN’s output.

Finally, the cross-correlated Markov DQN is used to classify the anxiety levels based on the extracted features and correlation analysis. The system determines whether the anxiety level is normal, mild, moderate, or severe. It utilizes the information gathered from the band cross-correlation analysis and the decision-making capabilities of the DQN to make accurate classifications. The network has learned patterns from the EEG data that are indicative of different levels of anxiety, allowing it to classify new instances of EEG data into appropriate categories.

Overall, with this proposed method, the harmonics in EEG data are eliminated and deep hidden temporal features are extracted, thereby improving the analysis of the impact of binaural beats on anxiety levels. Section 4 clearly explains the results and discussion of the proposed method.

## 4. Results and Discussion

This section includes a thorough discussion of the implementation results, as well as the performance of the proposed Denoised Harmonic Subtraction and Transient Temporal Feature Extraction method, and a comparison section to ensure that the proposed system successfully analyzes the impact of binaural beats on anxiety levels.

### 4.1. System Configuration

The developed system is implemented in MATLAB (version R2022a) and tested using a computer with the following technical characteristics:
SoftwareMATLABOSWindows 10 (64-bit)ProcessorIntel i5RAM8GB RAM

### 4.2. Simulated Output of the Proposed Model

The simulated output of the proposed model for Denoised Harmonic Subtraction and Transient Temporal Feature Extraction has been explained in this section from the initial setup.

**Figure 7:**
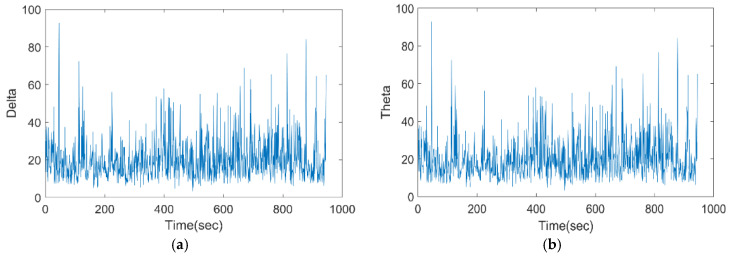

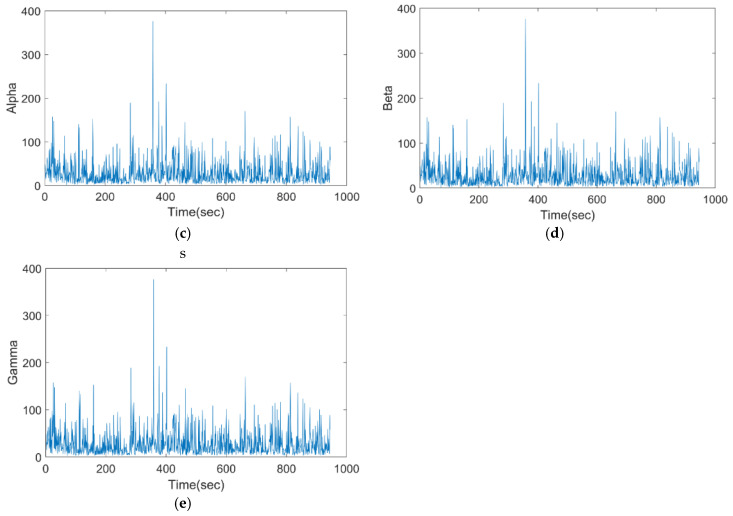
Input EEG of the proposed model for (**a**) delta, (**b**) theta, (**c**) alpha, (**d**) beta, and (**e**) gamma.

Figure 7 depicts the input EEG of the proposed model for varied times. It has been discovered that the corresponding EEG frequencies are modulated by delta, theta, alpha, beta, and gamma binaural beats. Delta and theta range from 0 to 100 Hz, and alpha, beta, and gamma range from 0 to 400 Hz. These data are sent to the Wiener fused convo filter for pre-processing.

**Figure 8:**
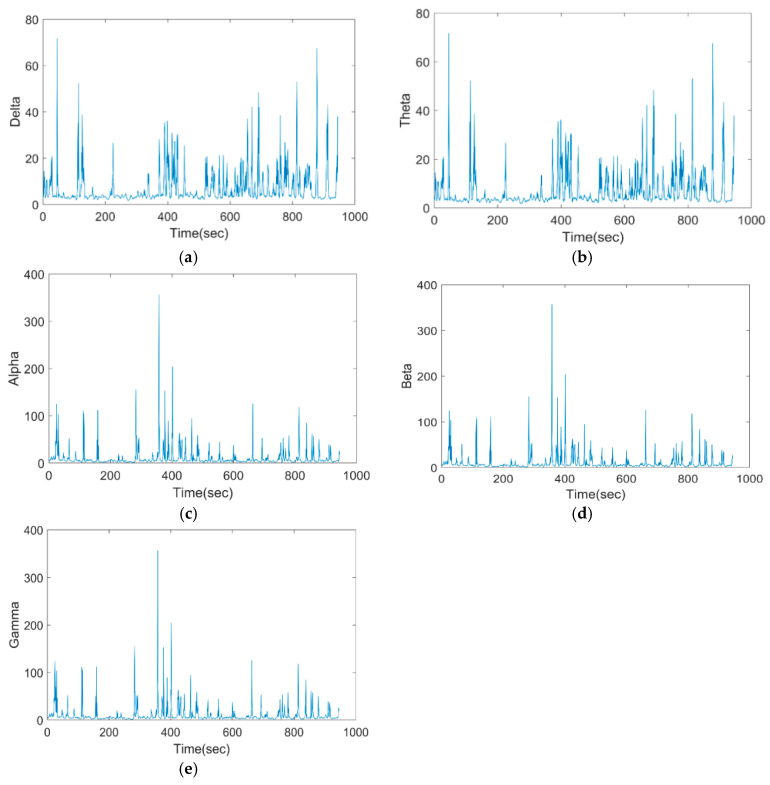
Pre-processed EEG of the proposed model for (**a**) delta, (**b**) theta, (**c**) alpha, (**d**) beta, and (**e**) gamma.

Figure 8 shows the pre-processed frequencies of the proposed model for various frequencies, such as delta, theta, alpha, beta, and gamma.

The Wiener Fused Convo Filter uses the CAE to capture complex spatial features like electrode-related noise in EEG signal components, and then merges with the Wiener filter to remove linear noise components.

**Figure 9:**
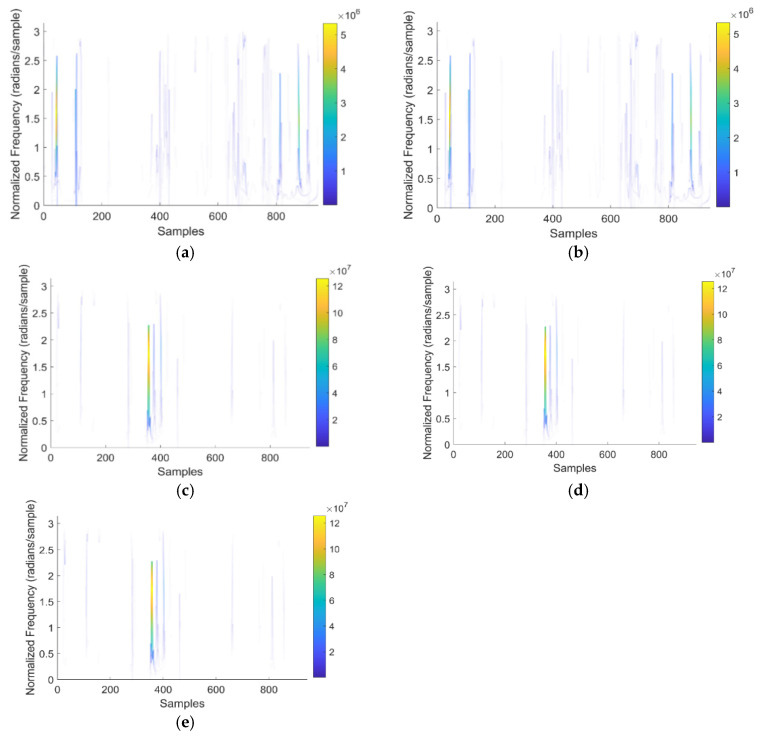
Normalized frequencies of the proposed model for (**a**) delta, (**b**) theta, (**c**) alpha, (**d**) beta, and (**e**) gamma.

Figure 9 depicts the normalized frequencies of the proposed model for various frequencies, such as delta, theta, alpha, beta, and gamma.

The HHT method decomposes EEG data into intrinsic mode functions and instantaneous frequencies, revealing non-linear oscillatory components. The decomposed data are processed by CNN to capture nonlinear summation and resonant coupling effects in the brain rhythm, preventing the misinterpretation of neural activity. As illustrated in Figure 9, normalization is applied to the EEG data before feature extraction and classification. This process ensures that the input data are scaled to a standard range, which is essential for several reasons: Normalization helps speed up the convergence of the training process for the DQN. By ensuring that all input features contribute equally to the learning process, the model learns more effectively and efficiently.

**Figure 10:**
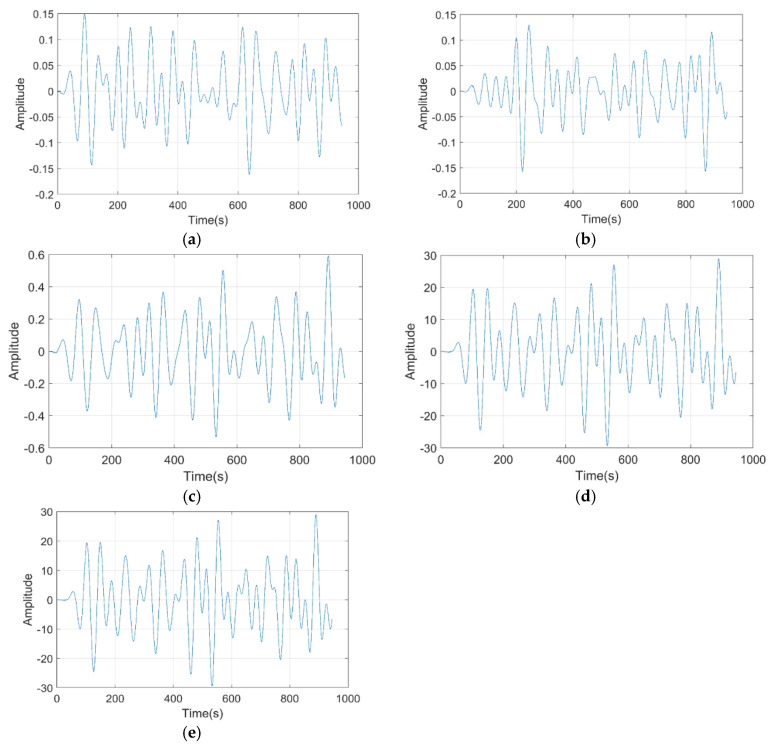
Brain rhythm of the proposed model for (**a**) delta, (**b**) theta, (**c**) alpha, (**d**) beta, and (**e**) gamma.

Figure 10 depicts the brain rhythm of the proposed model for various frequencies, such as delta, theta, alpha, beta, and gamma. The EEG data are initially embedded with relative vector positional encoding to capture the sequence of brain rhythm patterns in the temporal order, and then, these embedded EEG data are fed into the Transformer-XL Architecture, which uses a dynamic allocative attention mechanism to detect signal patterns related to transient events in the EEG data.

**Figure 11:**
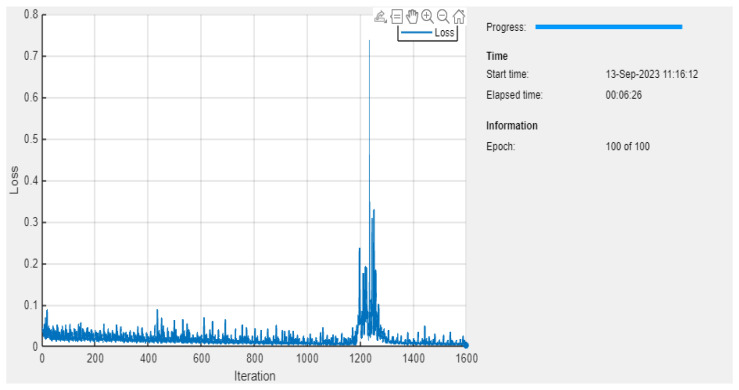
The loss rate of the proposed system.

Figure 11 shows the loss rate of the proposed system ranges from 0 to 0.7, approximately.

**Figure 12:**
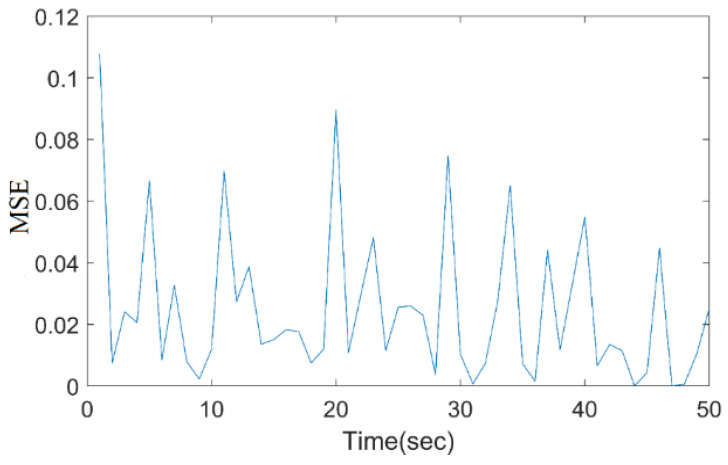
Mean square error (MSE) of the proposed model.

The very sharp and tall peak occurs just after the 1200th iteration mark, where the loss value spikes dramatically to just above 0.7, before falling back down. The sharp spike in the loss curve at iteration 1200 likely occurred due to a temporary gradient instability, causing the model to overshoot the optimal weights. Once the gradient adjustments stabilized, the loss value returned to a lower, more consistent range. Other than this peak, most of the loss values seem to be oscillating mostly below 0.1, with several smaller peaks throughout. Figure 12 depicts the MSE of the proposed system with a varied time period.

Here, the MSE ranges from 0 to 0.12, with sharp peaks and troughs. The MSE peaks near 0.1 within the first few seconds, then drops back to around 0.02 before rising again. This pattern continues with several peaks and troughs throughout the process.

**Figure 13:**
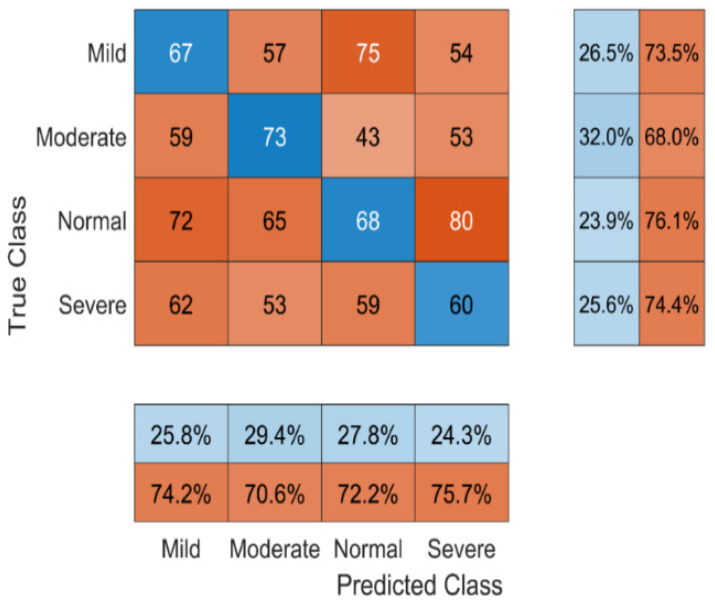
Confusion matrix of the proposed method.

Figure 13 illustrates the confusion matrix of the proposed system for four different classes, such as mild, moderate, normal, and severe.

A confusion matrix, sometimes referred to as an error matrix, is a table that shows how well a machine learning system for supervised classification performs. The ultimate accuracy of the suggested model on test data is 95.6%. Section 4.3 displays the performance metrics of the proposed system.

### 4.3. Performance Metrics of the Proposed System

**Figure 14:**
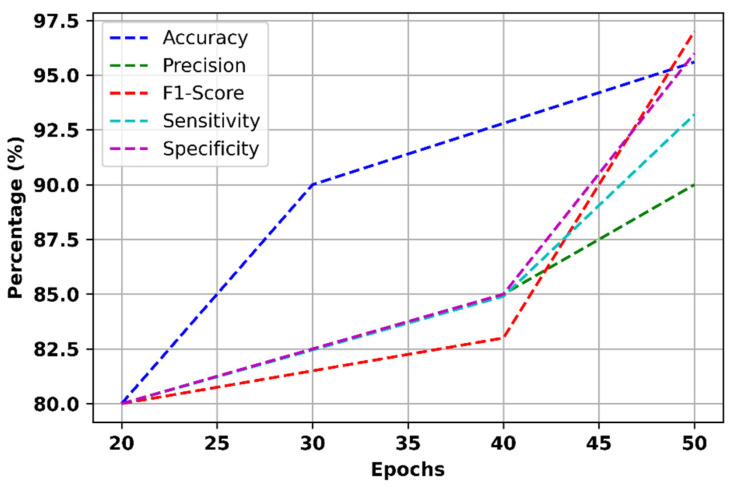
Accuracy, precision, sensitivity, specificity, and F1 score of the proposed model.

In this section, a detailed explanation of the effectiveness of the suggested technique and the achieved outcome were explained. Figure 14 displays several key metrics such as the accuracy, precision, sensitivity, specificity, and F1 score of the proposed model at various epochs.

The proposed model’s performance improves with increasing epochs, achieving its highest accuracy of 95.6% at 50 epochs, compared to a minimum of 80% at 20 epochs. This improvement is attributed to the cross-correlated Markov DQN classification model, which enhances the understanding of anxiety levels by accounting for the influence of various frequency bands and their temporal correlations. Similarly, the model’s precision peaks at 90% at 50 epochs and drops to 80% at 20 epochs, reflecting its ability to capture the temporal continuity of EEG data. The Embedded Transfo XL Recurrent Network plays a key role in improving precision by offering a deeper understanding of brain dynamics over time. The sensitivity of the model reaches its maximum of 93.2% at 50 epochs, with a minimum sensitivity of 83% at 30 epochs. This increase in sensitivity is due to the Wiener Fused Convo Filter and Intrinsic Harmonic Subtraction Network, which ensure that the extracted features more accurately reflect natural brain activity linked to anxiety levels. The specificity also improves, reaching a maximum of 96% at 50 epochs and a minimum of 84% at 35 epochs. This is due to the Intrinsic Harmonic Subtraction Network’s ability to eliminate irrelevant signal components, preserving the key features for accurate analysis. Finally, the F1 score, which measures the balance between precision and recall, reaches 87% at 50 epochs and drops to 80% at 20 epochs. The cross-correlated Markov DQN framework’s consideration of EEG signal dynamics at different frequencies and time intervals enables the model to better differentiate between anxiety levels, resulting in higher F1 scores at optimal epochs. This demonstrates the effectiveness of the proposed model in capturing both temporal and frequency-specific features of the EEG data, leading to an enhanced classification performance.

**Figure 15:**
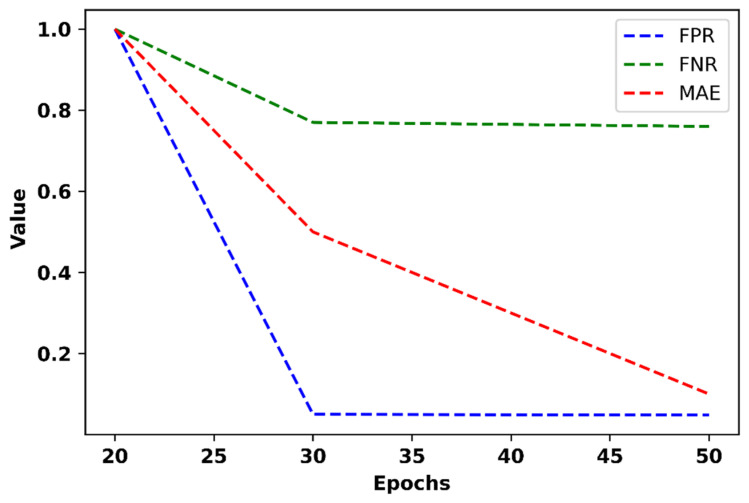
FPR, FNR, and MAE of the proposed model.

Figure 15 displays the FPR, FNR, and MAE of the proposed model at various epochs.

The proposed method demonstrates significant improvements in performance metrics as the number of epochs increases. At epoch 50, the model achieves the lowest FPR of 0.048, while the highest FPR of 1 is observed at epoch 20. This improvement is attributed to the Wiener Fused Convo Filter and Intrinsic Harmonic Subtraction Network, which effectively remove noise, ensuring that the extracted features are more reflective of true neural activity. Similarly, the FNR reaches its lowest value of 0.076 at epoch 50, compared to a maximum of 1 at epoch 20. The Intrinsic Harmonic Subtraction Network plays a key role in eliminating harmonic distortions, retaining only relevant neural dynamics, and minimizing the misinterpretation of EEG signals. The model also achieves a minimum MAE of 0.1 at epoch 50, while the maximum MAE of 1 occurs at epoch 20. The cross-correlated Markov DQN classification framework’s dynamic decision-making and band cross-correlation analysis help capture subtle differences in anxiety levels, thereby reducing the MAE. These results highlight the effectiveness of the proposed model in minimizing error rates and ensuring a more accurate classification of anxiety levels based on EEG data.

**Figure 16:**
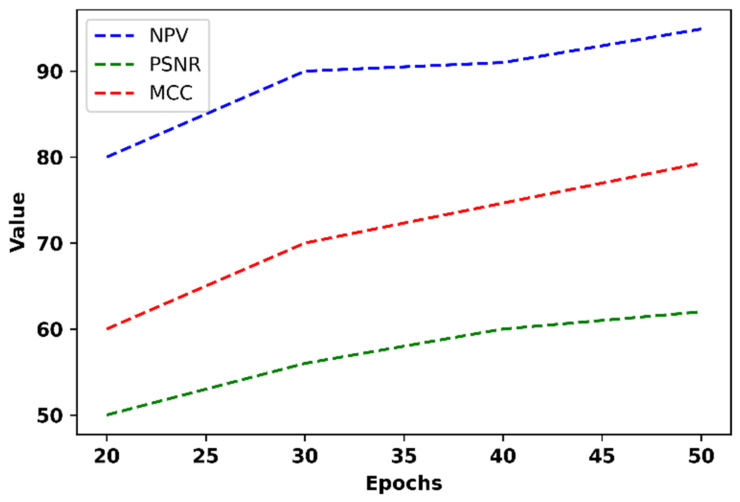
NPV, PSNR, and MCC of the proposed model.

Figure 16 displays the suggested model’s NPV, PSNR, and MCC at different epochs.

The proposed method achieves a high NPV of 94.9% at epoch 50, with the lowest NPV of 90% at epoch 30. The Transient Temporal Feature Extraction method plays a significant role in increasing the model’s sensitivity, allowing it to better detect true negatives and thus enhance the NPV. Regarding the MCC, the model reaches a maximum value of 79.3% at epoch 20, with the lowest MCC of 60% at epoch 50. The cross-correlated Markov DQN framework effectively captures the relationship between the anxiety levels and binaural beat frequencies, leading to higher MCC scores. Additionally, the PSNR of the proposed method improves with increasing epochs, achieving the maximum PSNR of 62 at epoch 50 and the lowest PSNR of 50 at epoch 20. This improvement is attributed to the Embedded Transfo XL Recurrent Network, which enhances signal fidelity by identifying and extracting relevant features. The overall performance, as demonstrated by these metrics, reflects the model’s ability to accurately capture and analyze the EEG signals related to anxiety levels. Section 4.4 will provide a detailed comparison of the proposed model with existing methods.

### 4.4. Comparison of Proposed Model with Previous Models

This section emphasizes the effectiveness of the proposed model by comparing it with the outcomes of existing methodologies and illustrating its outcomes based on several metrics. The comparisons are made from the previous techniques with varying accuracy, precision, sensitivity, specificity, F1 score, FPR, FNR, NPV, FDR, and MCC. Comparisons are made with the existing techniques such as Blind Source Separation-Support Vector Machines (BSS-SVM), Firefly–Levenberg–Marquardt (FLM), Independent Component Analysis and Adaptive Noise Cancellation (ICA-ANC), and Distance Sorted-Electric Fish Optimization and Deformable Convolutional Networks (DS-EFO-DCN) [29]. All reported performance metrics for the existing models were benchmarked against the same dataset and evaluation criteria used for the proposed model. This ensures a fair comparison and validity of the results. The metrics were obtained from previously published studies, and where applicable, we have reproduced the results using the original authors’ methodologies to confirm their accuracy. This rigorous benchmarking process allows us to demonstrate the improvements offered by this proposed model to established techniques.

**Figure 17:**
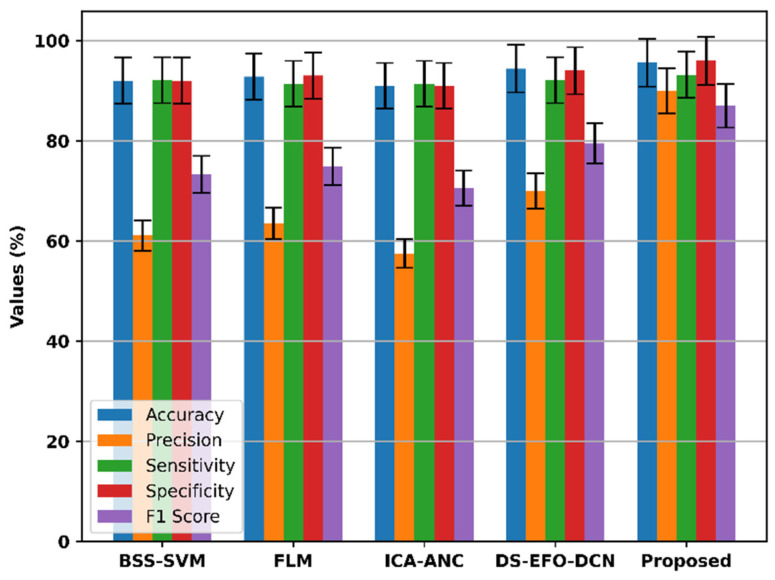
Comparison of key metrics such as accuracy, precision, sensitivity, specificity, and F1 score of the proposed model.

Figure 17 compares the performance of the proposed model with several existing models, including BSS-SVM, FLM, ICA-ANC, and DS-EFO-DCN, across key metrics such as the accuracy, precision, sensitivity, specificity, and F1 score.

Current models, including BSS-SVM, FLM, ICA-ANC, and DS-EFO-DCN, exhibit accuracies of 92%, 92.8%, 91%, and 94.4%, respectively. Compared with existing techniques, the proposed model achieves a higher accuracy of 95.6%. In terms of precision, the existing models, such as BSS-SVM, FLM, ICA-ANC, and DS-EFO-DCN, achieve a precision of 61.1%, 63.5%, 57.5%, and 70%, respectively. With a high precision rate of 90%, the suggested model outperforms current methods. Existing models such as BSS-SVM, FLM, ICA-ANC, and DS-EFO-DCN have sensitivity values of 92.1%, 91.4%, 91.4%, and 92.1%. The proposed model exceeds existing techniques with a high sensitivity rate of 93.2%. The specificity values of BSS-SVM, FLM, ICA-ANC, and DS-EFO-DCN are 92%, 93%, 91%, and 94% for existing models. The suggested model outperforms current methods with a high specificity rate of 96%. For the models that are currently in use, such as BSS-SVM, FLM, ICA-ANC, and DS-EFO-DCN, the F1 score values are 73.3%, 74.9%, 70.5%, and 79.5%. With a high F1 score rate of 87%, the proposed model performs better than the existing models. These results highlight the effectiveness of the proposed model in classifying anxiety levels based on EEG data, demonstrating its superiority over current techniques in key performance areas.

**Figure 18:**
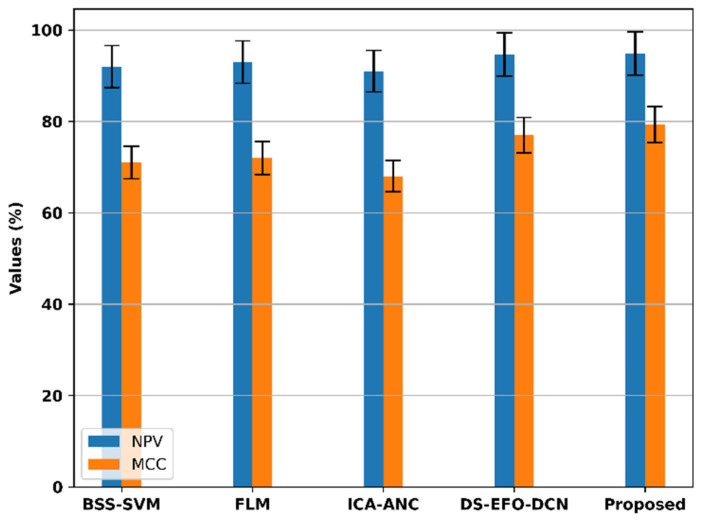
Comparison of the NPV and MCC of the proposed model.

The NPV and MCC of the proposed model and the existing models are compared in Figure 18.

The proposed model outperforms existing models in terms of key performance metrics, including the MCC and NPV. The existing models, BSS-SVM, FLM, ICA-ANC, and DS-EFO-DCN, have NPV values of 92%, 93%, 91%, and 94.7%, respectively. The proposed model outperforms the existing models with a higher NPV rate of 94.9%. For the MCC metric, the current models BSS-SVM, FLM, ICA-ANC, and DS-EFO-DCN have values of 71%, 72%, 68%, and 77%, respectively. The proposed model achieves a significantly higher MCC rate of 79.3%, demonstrating its superior performance compared to the existing techniques. These results highlight the effectiveness of the proposed model in providing more reliable and accurate assessments compared to existing approaches.

**Figure 19:**
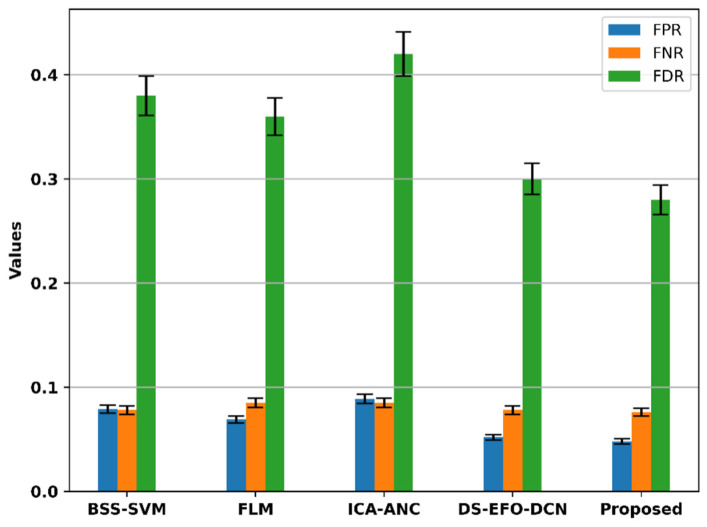
Comparison of the FNR, FPR, and FDR of the proposed model.

Figure 19 shows a comparison between the FNR, FPR, and FDR of the current models and the suggested model.

The figure also provides additional performance metrics for the EEG signal processing models. For the existing models, the FPR values are 0.079 for BSS-SVM, 0.069 for FLM, 0.089 for ICA-ANC, and 0.052 for DS-EFO-DCN. The proposed model outperforms the current models with a lower FPR of 0.048. Regarding the False Negative Rate (FNR), the existing models BSS-SVM, FLM, ICA-ANC, and DS-EFO-DCN have values of 0.078, 0.085, 0.085, and 0.078, respectively. The proposed model achieves a lower FNR of 0.076 compared to the current techniques. Additionally, the FDR for the existing models BSS-SVM, FLM, ICA-ANC, and DS-EFO-DCN are 0.38, 0.36, 0.42, and 0.3, respectively. The proposed model demonstrates a superior performance with a lower FDR of 0.28.

Overall, in the results section, the proposed model is compared to existing models, and the performance is explained using graphs. This demonstrates that the novel Denoised Harmonic Subtraction and Transient Temporal Feature Extraction has a high accuracy of 95.6%, precision of 90%, sensitivity of 93.2%, specificity of 96%, F1 score of 87%, NPV of 94.9%, and MCC of 79.3%, and low FPR of 0.048, FNR of 0.076, and FDR of 0.28 when compared to the previous models.

## 5. Conclusions

In conclusion, the proposed method presents a comprehensive approach to address the limitations of existing EEG signal processing techniques for assessing the impact of binaural beats on anxiety levels. By introducing innovative denoising techniques and harmonic subtraction networks, the system effectively eliminates noise and misinterpretation, while retaining relevant neural dynamics. The Embedded Transfo XL Recurrent Network further enhances the analysis by capturing temporal continuity and identifying transient event patterns in EEG data, including rapid oscillatory bursts. Moreover, the cross-correlated Markov DQN classification framework enables a robust assessment of anxiety levels, considering the influence of different frequency bands. This integrated approach facilitates a deeper understanding of the relationship between binaural beats and anxiety, offering potential insights for therapeutic interventions. Thus, the results of the simulation demonstrated that the proposed model has a high accuracy of 95.6%, precision of 90%, sensitivity of 93.2%, specificity of 96%, F1 score of 87%, NPV of 94.9%, and MCC of 79.3%, and low FPR of 0.048, FNR of 0.076, and FDR of 0.28 when compared to the previous models. Overall, the proposed method demonstrates significant advancements in EEG signal processing, enhancing the accuracy and reliability of anxiety level classification in the context of binaural beat interventions. The integration of the Wiener filter, CNN, Transformer-XL, LSTM, and DQN enhances the model’s performance but introduces significant computational challenges. The complexity of these networks increases processing time and resource consumption, especially with large datasets or real-time applications. To mitigate these issues, optimization strategies like model pruning, quantization, and smaller network versions need to be employed. Additionally, batch processing and efficient memory management helps manage resource demands on limited hardware. This makes the model feasible even on less powerful hardware, while retaining its effectiveness. Utilizing cloud-based platforms for training and inference alleviates hardware limitations, providing access to more powerful computational resources.

## Data Availability

The original contributions presented in the study are included in the article, further inquiries can be directed to the corresponding authors.
